# Pyrroles as a Potential Biomarker for Oxidative Stress Disorders

**DOI:** 10.3390/ijms24032712

**Published:** 2023-02-01

**Authors:** Brett Lambert, Annalese Semmler, Cristina Beer, Joanne Voisey

**Affiliations:** 1 Applied Analytical Laboratories, Logandowns Dr, Meadowbrook, QLD 4131, Australia; 2 School of Clinical Sciences, Faculty of Health, Queensland University of Technology, Kelvin Grove, QLD 4059, Australia; 3 Centre for Genomics and Personalised Health, School of Biomedical Sciences, Faculty of Health, Queensland University of Technology, Kelvin Grove, QLD 4059, Australia

**Keywords:** pyrroles, urobilinogen, schizophrenia, biomarkers, psychosis, treatment response, diagnosis, oxidative stress, mental health, psychiatric disorders

## Abstract

Redox imbalance or oxidative stress that results from both environmental and genetic factors is observed in patients with schizophrenia. Therefore, identifying markers of oxidative stress in the early stages of psychosis and using antioxidant treatments as an adjuvant to antipsychotics has important implications. The reaction of *p*-*N*,*N*-dimethylaminobenzaldehyde (DMAB) with pyrrole moieties has been well studied for well over a century for use as a marker of oxidative stress dysregulation. Throughout this time, pyrroles have been investigated with varying veracity in urine extracts to identify elevated levels in patients diagnosed with schizophrenia. Since the 1960’s, various claims have been made with respect to what causes the colour change when DMAB is added to urine extracts. Whilst the substances from this reaction have not been fully elucidated, an objective look at most studies indicates that urobilinogen is likely to be one them. Urobilinogen has also been identified as a major interferent in our results. Both pyrroles and urobilinogen condense the DMAB reaction system (form condensation products) and are quite different. The urobilinogen detected in urine forms when gut microflora chemically reduces the bilirubin content of bile acids. In comparison, evidence suggests that the pyrrole fraction originates from the fragmentation of regulatory haem by reactive oxygen species (ROS) such as hydrogen peroxide and super and nitrous oxides. Clinical studies in our laboratories have established that pyrroles as a urine biomarker have specificity in detecting schizophrenia; however, caution must be applied as the readings are subject to interference by other DMAB active compounds that are present, such as urobilinogen. This review highlights the initial chemistry in isolating pyrroles and provides recommendations for standardised laboratory testing to ensure pyrroles are correctly measured and distinguished from other by-products.

## 1. Pyrroles

**Figure 1:**
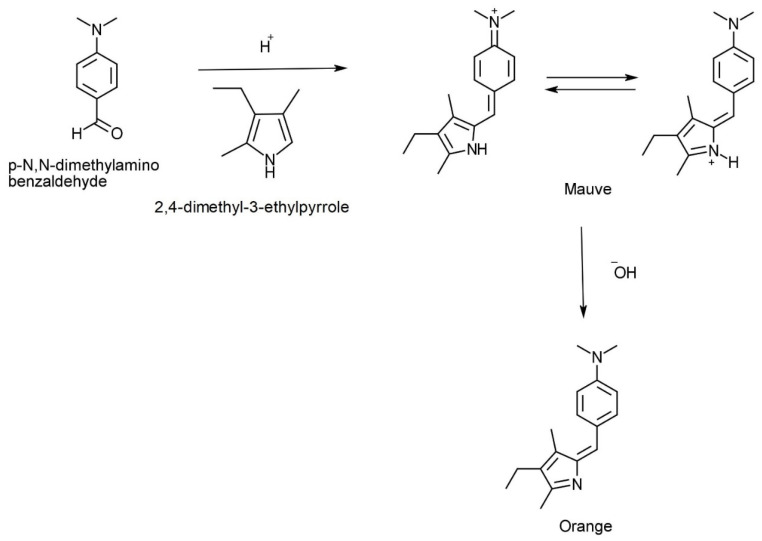
The reaction of *p*-*N*,*N*-dimethylamino benzaldehyde with a model pyrrole (this sequence has been confirmed by NMR in our laboratories).

Pyrrole was first studied comprehensively in 1938 as a doctoral thesis [1] and originates from the Greek word meaning fiery red. It was initially discovered in 1932 when pine needles dipped in hydrochloric acid turned red following exposure to vapour containing pyrroles [2]. This reaction is now understood as the combination of *p*-*N*,*N*-dimethylaminobenzaldehyde (DMAB) with the pyrrole moiety (Figure 1). Pyrroles (a class of five-membered nitrogen containing heterocycles) can be found as chemical sub-units in porphyrin systems such as vitamin B_12_, bile pigments (biliverdin and bilirubin), haem, and chlorophylls [3].

It was not until decades later that a substance, supposedly identified as “kryptopyrrole” and then later hydroxy-haemopyrrolenone (HPL) [4], was detected in urine and associated with psychiatric disorders such as schizophrenia, anxiety, and depression [5]. Mauve factor is another term used for pyrrole and kryptopyrrole [6], a term coined to reflect the mauve-coloured complex that forms when active pyrrole derivatives react with DMAB in urine extracts.

We propose here that most of the past urine testing for pyrroles has detected urobilinogen (as has been characterised by spectroscopy and confirmed by interference studies in our laboratory, including pure urobilinogen spike recovery experiments). This ambiguity is likely because pyrroles and urobilinogen react similarly with DMAB (forming mauve-coloured complexes albeit with different wavelength (lambda) max (λ_max_) values (Table 1)).

We hypothesise that pyrroles become elevated when physical and psychological stress results in excess production of peroxides (the markers of oxidative stress). This process is referred to as an “oxidative burst” and subsequently leads to an increase in the fragmentation of regulatory haem and the accumulation of pyrroles. As mentioned earlier, the by-product urobilinogen is formed in an entirely different manner. As psychiatric disorders are associated with markers of oxidative stress, it becomes important to differentiate between the two different by-products. This is especially important when diagnosing psychosis, as patients with bile disorders can present with similar aetiology as psychiatric disorders. Psychiatric disorders are complex and can present with many comorbidities that are often associated with poor physical health [7,8]. Management of bile-related disorders compared with oxidative stress disorders is also quite different, so to ensure appropriate patient management it is important to distinguish between the two different pathophysiologies.

The method established in our laboratory physically separates the water-soluble pyrroles, such as zwitterionic porphobilinogen (PBG), and spectrophotometrically distinguishes between urobilinogen and the reactive pyrrole fraction. Both by-products can be measured independently. We have found that quantitation of the urobilinogen level can be useful in that it gives an indication of bile flow and renal function. The absence of urobilinogen is indicative of bile flow obstruction (e.g., gall stones and splenic infection), while elevated urobilinogen, at a measure greater than 16–33 umol/L, is indicative of kidney injury or impaired renal function. Measuring pyrrole levels is used as a marker of the level of oxidative stress or regulatory haem damage.

## 2. Previous Clinical Studies with Pyrroles

As reviewed by Warren et al. (2021), numerous studies have attempted to correlate elevated pyrrole levels with the diagnosis of psychiatric disorders but have had limited effect [9]. Unfortunately, most of these studies did not use statistical analyses and/or control group data or had limited sample sizes, which has limited the validity of the data [5,6,10,11,12,13,14,15,16,17]. Only the Fryar-Williams study followed the “Lambert” method guidelines for collections with results being corrected to creatinine to adjust for hydration [18]. The “Lambert” method (Applied Analytical Laboratories, Meadowbrook, Australia) has also established that a labile haem pyrrole (“expressed as HPL”) has merit as a biomarker for case detection of schizophrenia and/or schizoaffective psychosis. This work clearly demonstrated that urinary pyrrole concentration had a significant statistical correlation with patient symptoms as assessed by the modified DSM-(IV), with overall diagnosis detection sensitivity of 70% and specificity of 64% [18]. Oxidative stress is well documented in schizophrenia [19,20,21], and the Lambert method [14,18,22] suggests that the determination of urinary pyrrole concentration has value as a non-invasive oxidative stress biomarker that, when used as part of an array of tests and clinical assessments, provides significant improvement in diagnostic accuracy for psychiatric conditions and therefore potentially improves treatment outcomes for patients. In previous studies, we also identified genetic and epigenetic markers in *DTNBP1* and *DISC 1* that are associated with both schizophrenia and oxidative stress [7,23,24,25]. It is likely that alterations in genetic and environmental factors result in redox dysregulation, which thus appears as elevated pyrroles in the urine. As mentioned in the recent review by Rambaud et al., oxidative stress is not only linked to schizophrenia, but also to neurological and metabolic disorders [21]. Therefore, it is important to use oxidative stress markers, such as pyrroles, in conjunction with clinical, biochemical, and genetic markers for disease diagnosis.

## 3. Chemistry of HPL, DMAB, and Pyrroles

There have been studies that have investigated the chemistry of HPL, DMAB, and pyrroles; however the data remain controversial. There is a vast amount of anecdotal commentary suggesting that B6 and pyrroles form a complex. Some groups have published chemical structures of such (without spectroscopic data such as NMR chemical shift data to confirm) and the veracity of these hypotheses is doubted. Interestingly Van Urk’s paper regarding the reaction of a series of organic compounds (135 mostly medicinal products) with acid-activated *DMAB* showed a distinct reaction with pyrrole and indole derivatives and no reaction with pyridine derivatives [26]. This is unusual as pyridoxal is a pyridine derivative. To verify this, we reproduced the conditions favourable for a DMAB reaction with 3-ethyl-2,4-dimethyl pyrrole and the pyridoxal vitamer of B_6_ (substituting aldehydic DMAB for aldehydic pyridoxal) and monitored reaction progress using UV-Vis and LC-MS without success. This suggests that the premise that pyridoxal undergoes a quench by pyrrolic substances in vivo is weak.

**Figure 2:**
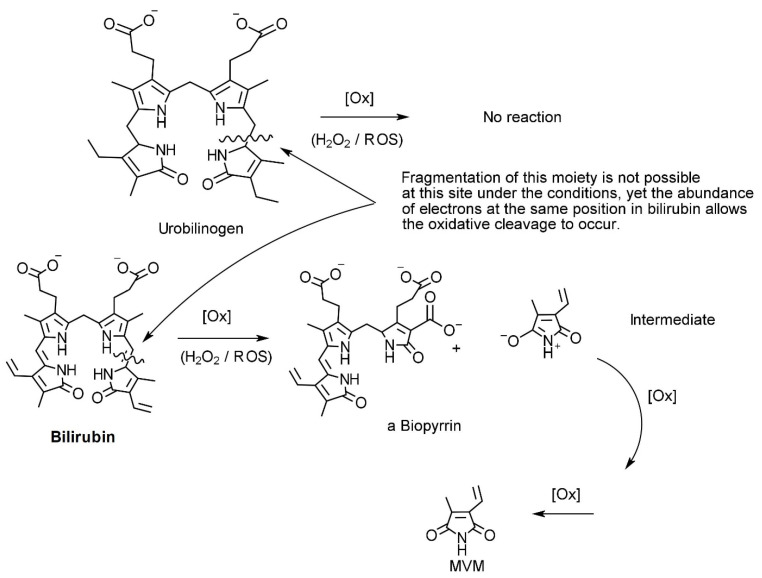
Potential pathways for oxidation of urobilinogen and bilirubin by reactive oxygen species (ROS) such as peroxide.

More recently, studies describing the detection of bilirubin oxidation products in the serum and urine of patients following subarachnoid haemorrhage (SAH) using an Enzyme Linked Immunosorbent Assay (ELISA) technique have been published [27,28,29]. The detected materials from the ELISA technique resemble the remaining cleaved fragment in our urine samples. The symptomology also mirrors that observed in cases of elevated urinary pyrrole (see Figure 2).

As part of a National Association of Testing Authorities (NATA) technical review (2018), we undertook several studies to characterise what was being measured in urine and to ensure that pyrroles were measured independently of urobilinogen. To account for total DMAB and urobilinogen activity in the samples, parallel urine dipstick analysis was performed using an automated Siemens Advantus 10SG system. Known concentrations of DMAB-reacted urobilinogen were used as external references in the AAL assay and DMAB-reacted 3-ethyl-2,4-dimethyl pyrrole was used to calibrate the assay.

The results of our technical review reinforced that that the origin of our reported “oxidative stress marker” pyrrole is not urobilinogen, as there are not enough electrons at the relevant/required positions of cleavage for the corresponding fragmentation to occur (Figure 2). However, there are enough in regulatory haem species such as biliverdin or bilirubin (remembering as a “rule of thumb” that carbon–carbon double bonds are easier to break than carbon–carbon single bonds).

More recently, we have focused on the photo-oxidation chemistry of 3-ethyl-2,4-dimethyl pyrrole to understand the chemistry occurring between the carbon at the alpha position and the nitrogen atom of the ring structure. This work is on-going and aims to further elucidate the chemical mechanism behind the oxidative process, with a further aim of paving the way for more effective targeted treatments.

## 4. Treatment of Elevated Pyrroles

It is well understood that vitamin B_6_ does not bind to pyrroles due to the incompatibility of the relevant chemistries. Based on the previous observations, some researchers have suggested treatments for patients with elevated levels of pyrroles that include supplementation with active B_6_ and zinc. Clinical trials performed by Pfeiffer et al. first identified this link, demonstrating that supplementation of B_6_ and zinc led to significant reductions in DMAB-active pyrrole and improvement in patient symptoms [12]. According to clinicians in this field, ongoing treatment of this nature is required to reduce or suppress symptoms [30,31]. However, this has not been without harm, as numerous cases of B_6_ toxicity have been recorded (Recent Therapeutic Goods Authority B6 Warning) which again highlights the need for better understanding of the biochemistry involved. It is possible that the potential benefit of B_6_ treatment may be due to its antioxidant properties. However, further research is needed to identify other antioxidants, such as N-acetyl cysteine, that may be equally or more effective without the side-effects of accumulation [21].

## 5. Limitations of the Assay

**Figure 3:**
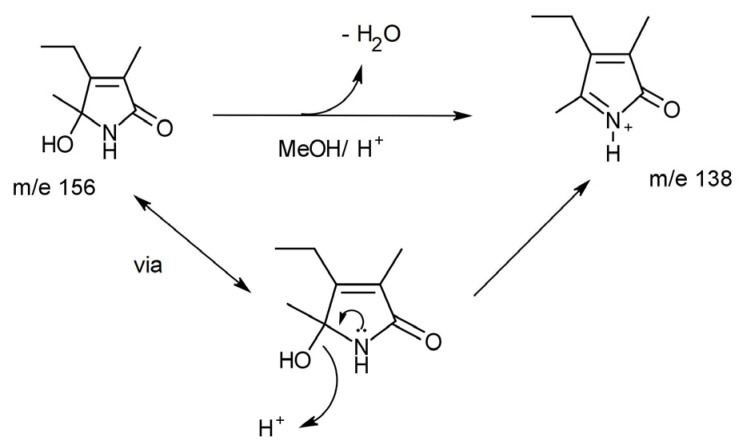
The hydroxylactam of haem pyrrole (“HPL”, m/e156) dehydroxylates under mildly acidic conditions (such as those encountered in typical LC-MS) to the m/e ion shown above.

There are several limitations with this assay. The first is the fact HPL or pyrroles have been confused with urobilinogen, which has clouded the clinical picture. This is also confirmed in the review conducted by Warren et al. that established that few clinical studies could confirm an association between elevated HPL and mental health symptoms [9]. Numerous studies have not used controls, which thus impacts the validity of their data. From those that have used controls, they have reported large standard error margins between the positive samples and the controls. From our recent work (not yet published), it is likely that the published pyrrolinone structure for “HPL” is incorrect, and quantitative measurement by LC-MS is probably limited by the detection of an artefact produced by the reactive pyrrole as it runs through the assay. To support this, there is evidence that these compounds re-arrange rapidly under acidic conditions. The conditions used for LC-MS are sufficiently acidic (pH 3–4) to significantly change the chemistry of the specimen, thus potentially generating the artifact and resulting in the large standard error margins and significant overlap between “positive cases and controls” as quoted recently [32]. Figure 3 demonstrates the rearrangement we observed that pure HPL undergoes in LC-MS conditions de novo.

Due to the diverse nature of the corresponding chemistry, there are also likely to be any number of interchangeable chemical structures with similar molecular masses emerging from the LC-MS process. Therefore, the currently “accepted” treatment recommendations for elevated pyrrole (which include high doses of vitamin B_6_) should be approached with caution. Another limitation is that pyrrole is not a single definitive marker of mental health and should be used in conjunction with other clinical biochemistry measures and clinical assessments where its efficacy is significantly improved [18].

## 6. Recommendations

Our evidence shows that the material being detected has resulted from a biological/physiological event (oxidative burst) in which a DMAB-reactive intermediate is generated that is highly reactive yet potentially stable enough to remain intact in the bladder in sufficient levels to be measured. This then rearranges to resemble 5-hydroxy-3-ethyl-4,5-dimethylpyrrolen-2-one (or an analogue). From studying the kinetics of degradation for control samples, we know that the degradation displays 2nd to 3rd order kinetics, which confirms that each molecule of our biochemical source generates or releases between two and three DMAB-active analyte molecules (even when correcting for urobilinogen). This points towards and reinforces the suggestion that the molecular origin is regulatory haem.

More recently, our focus on the photo-oxidation chemistry of 3-ethyl-2,4-dimethyl pyrrole has led us to understand the chemistry occurring between the carbon at the alpha position and the nitrogen of the ring structure. This work is aimed at developing an understanding of the chemical mechanism of the oxidative stress process to pave the way for more effective targeted treatments for applicable psychiatric conditions.

The information presented here highlights the importance of standardised laboratory testing to ensure HPL is correctly measured and distinguished from other by-products, such as urobilinogen. Whilst further research that uses control samples and statistical analyses is required to validate the association of elevated pyrroles with psychiatric symptoms, a validated tool such as the one we describe would be the first critical step in completing this research. An understanding of the role of biochemistry and biomarkers has the promise of an objective scientific method that can aid the diagnosis and treatment of psychiatric disorders.

## Figures and Tables

**Table 1:** Lambda (λ)_max_ values for the complexes formed between DMAB and various pyrrole moieties. These values were determined by AAL using a GBC Cintra 2020 double beam UV-Vis NIR spectrophotometer.

Compound Reacted with DMAB	Lambda Max (nm)
2,4-dimethyl pyrrole 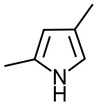	540
3-ethyl-2,4-dimethyl pyrrole 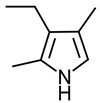	538
2,5-dimethyl pyrrole 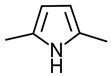	525
Urobilinogen 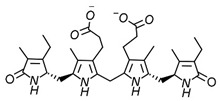	2 peaks: 475 and 560
Bilirubin 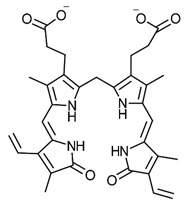	2 peaks: 440 and 509
